# Perioperative diagnostics of patients referred for radioiodine therapy of differentiated thyroid carcinoma: referral center experience in an iodine-insufficient country

**DOI:** 10.1007/s12020-020-02509-9

**Published:** 2020-11-01

**Authors:** Friederike Eilsberger, R. Michael Tuttle, Damiano Librizzi, Andreas Pfestroff, Markus Luster, Frederik A. Verburg

**Affiliations:** 1 Department of Nuclear Medicine, University Hospital Marburg, 35043 Marburg, Germany; 2 Department of Endocrinology, Memorial Sloan-Kettering Cancer Center, New York, NY USA; 3 Department of Radiology and Nuclear Medicine, Erasmus MC, Rotterdam, The Netherlands

**Keywords:** Differentiated thyroid cancer, Thyroidectomy, Thyroid ultrasound, Thyroid scintigraphy, Fine-needle biopsy

## Abstract

**Purpose:**

The interdisciplinary “Martinique-Principles” of four international professional societies concerned with the patient management of differentiated thyroid cancer (DTC) patients were agreed upon. Differences in perioperative diagnostics can lead to differences in clinical decision founding regarding the treatment of thyroid carcinoma. Our aim was to analyze the perioperative diagnostics of patients referred for postoperative I-131 therapy of DTC.

**Methods:**

We retrospectively examined the data of 142 patients who were referred to our center for the first course of postsurgical I-131 therapy. We extracted data on perioperative diagnostics.

**Results:**

Fine-needle biopsy (FNB) was performed in 27/142 patients. In 17 patients, FNB yielded findings suspicious of malignancy, in 3 patients a follicular lesion was reported. An intraoperative frozen section analysis was performed in 79/142 patients. 5/63 patients showed already a cytologically proven malignancy. In 10/79 patients, the frozen section had a nonmalignant result, although DTC was found on final assessment. In 2/79 patients, frozen section analysis was indecisive, although the final report confirmed DTC. In the remaining 67 patients, frozen section yielded DTC.

**Conclusions:**

There is room for improvement in perioperative diagnostics surrounding thyroid surgery, currently many procedures are performed without adequate information on potential presence of thyroid cancer. More frequent use of FNB might be able to decrease the number of unnecessary thyroid surgeries, increased use of frozen section might decrease the number of second operations and might contribute to less discordance between experts in the field of DTC treatment.

## Introduction

Generally, the recommended diagnostic cascade resulting in a diagnosis of differentiated thyroid cancer (DTC) involves neck ultrasound and fine-needle biopsy of any suspicious nodule. This is, depending on the results, followed by diagnostic or therapeutic thyroid surgery, with or without intraoperative frozen section analysis of suspicious nodules.

The surgical procedure is heavily influenced by the known diagnosis at the time of the procedure. Generally speaking, if thyroid cancer is known prior to surgery, the surgical approach will be oriented more strongly toward expandation total thyroidectomy versus lobectomy, extent of lymph node dissection, and possibly even increasing the likelihood that frozen section may be used intraoperatively, whereas the prevention of complications will be of paramount importance for surgery of supposedly benign disease.

In Germany, a (formerly) iodine-deficient country [1], the prevalence of nodular goiter is high. Consequently, in clinical practice, patients are often referred for surgery due to mechanical complaints. However, in a majority of patients referred for thyroid surgery no fine-needle biopsy, even of sonographically or scintigraphically suspicious nodules, is performed [2, 3].

Conceivably, the surgical procedure, especially with respect to radicality of thyroid resection, can be of consequence for further treatment of thyroid cancer. It has already been shown in literature that the benefit of postoperative I-131 therapy is variable, with some centers reporting no influence of outcome whereas other studies even show a benefit in microcarcinoma patients. Thus far, it is unknown whether the extent of surgical resection really differs between centers. Indeed, even the data surrounding differences regarding perioperative diagnostics (e.g. frequency of fine-needle biopsy or percentage of patients diagnosed by intraoperative frozen section) between centers in patients eventually diagnosed with thyroid cancer are scarce if not non-existent. At the recent intersocietal meeting of the European Association of Nuclear Medicine, the Society of Nuclear Medicine and Molecular Imaging, the European Thyroid Association, and the American Thyroid Association, it was postulated, that such diagnostic differences may well contribute a large part of the differences in I-131-related therapy choices between centers; it was suggested to perform further studies into this issue [4].

In a first attempt to answer this complex question, the aim of the present study was to analyze the perioperative diagnostics of patients referred for postoperative I-131 therapy of DTC in a tertiary referral center in a (formerly) iodine-deficient country.

## Materials and methods

### Patients

We retrospectively examined the data of all 146 patients who were referred to our tertiary referral center for the first course of postsurgical I-131 therapy of DTC between July 1, 2017 and June 30, 2019. Ethical approval and consent of the participants was not required for the study. Patients who only received lobectomy and were thus not referred for I-131 therapy were not included in this study. In four patients, insufficient data on preoperative diagnostic procedures were available for the present analysis; these patients were therefore excluded. In 17/142 (12%) patients, thyroid cancer was detected by FNB before surgery, in 67/142 patients (47%), frozen section yielded DTC. Details of the final study population of 142 patients are given in Tables 1–4.

**Table 1:** Basic characteristics of the study population

Characteristic	No. of patients or value as median (range)
Sex	
Female	101
Male	45
Median age (years)	
51 (14–86)	
Tumor histology	
Papillary thyroid cancer	131
Follicular thyroid cancer	7
Hürthle cell carcinoma	4
Poorly differentiated carcinoma	3
Insular thyroid cancer	1

**Table 2:** TNM classification of the study population

	N+	N0	No lymphnodes removed		M1
T1a	13	6	8	27	1
T1b	16	17	18	51	
T2	14	16	11	41	1
T3	6	7	8	21	1
T4	1	1		2	
	50	47	45	142	3

**Table 3:** T-stadium of the cases in which the indication for thyroid surgery was Graves’ disease, autonomously functioning thyroid nodule or goiter

T1a	T1b	T2	T3	T4
*n* = 5	*n* = 6	*n* = 4	*n* = 6	*n* = 0

*n* = number

**Table 4:** Study population classified by ATA risk of persistent/recurrent disease

ATA 1 (low risk)	*n* = 99
ATA 2 (intermediate risk)	*n* = 21
ATA 3 (high risk)	*n* = 22

*n* = number

### Data extraction

From our clinical files, we extracted data on perioperative diagnostics, including the patients’ history with specific complaints, clinical examination, neck ultrasound and, if available, thyroid scintigraphy with Tc-99m pertechnetate and/or Tc-99m MIBI as well as results of any fine-needle biopsy that was performed. Furthermore, we extracted data on the surgical procedure including the intraoperative findings as well as any frozen section that was performed. If a procedure such as e.g., frozen section was not explicitly reported, it was assumed not to have been done.

### Analysis

For the present study, data and results of presurgical diagnostics were employed as recorded in the attending physicians’ reports. As ultrasound reports were often scarce on details of suspicious nodules and did not report any TIRADS (or similar) classification, we did not endeavor to further stratify ultrasound findings other than the notion—as stated by the attending physician—of a sonographically suspicious nodule. Histological data were employed as recorded in the written pathologists’ report. Clinical staging of the patients was amended by data from post-I-131-therapy whole-body scintigraphy.

Data are reported descriptively either as numbers or percentage value.

## Results

### Preoperative diagnostics

Clinical examination and neck ultrasound were performed in all patients. 14/142 (10%) patients complained of obstructive or compressive symptoms in the neck.

In 123/142 (87%) patients, ultrasound revealed at least one suspicious nodule.

In 84 patients, thyroid scintigraphy was performed with Tc-99m pertechnate, yielding a hypofunctional nodule in 75/142 patients. In ten patients, additional thyroid scintigraphy was performed using Tc-99m MIBI, which yielded a suspicious finding in nine patients.

FNB was performed in 27/142 (19%) patients. In 17 patients, FNB yielded findings suspicious of malignancy, and in 3 patients a follicular lesion was reported. The remaining FNB results (7/142 (5%) patients) were not indicative of malignancy. Thus, in 17/142 (12%) patients, thyroid cancer was diagnosed before surgery.

### Indication for surgery

An overview of the indications for performing thyroid surgery is presented in Table 5. It is remarkable to note that 24 patients were operated upon based on ultrasound only and that in 56 patients, surgery was performed based on the presence of a scintigraphically hypofunctional thyroid nodule. Furthermore, five additional patients were treated surgically because of positive Tc-99m MIBI scintigraphy. However, in contrast to the general recommendation give in guidelines, in none of these aforementioned patients the suspicious imaging was followed by an FNB. In contrast, in only 20 patients, the result of a fine-needle biopsy was the basis for indicating thyroid surgery.

**Table 5:** Main indications for thyroid surgery

Characteristic	No. of patients
Suspicious neck ultrasound	24
Hypofunctional nodule in Tc-99m pertechnetate scintigraphy	56
Positive nodule in Tc-99m MIBI scintigraphy	5
Suspicious cytology	20
Graves’ disease	7
Autonomously functioning thyroid nodule	4
Goiter	10
Other reasons (e.g., lateral neck cyst)	16
No data	4

In 16 cases, the primary indication for a surgical procedure was provided by other, primarily non-thyroidal reasons, including primary hyperparathyroidism during which a concurrent removal the thyroid was decided preoperatively in one case and intraoperatively based on frozen section in another case, removal of a lateral neck cyst, which on frozen section was proven to be a metastasis of DTC, or known lymph node metastases removed in a prior surgical procedure.

### Intraoperative frozen section

An intraoperative frozen section analysis was performed in 79/142 (47%) patients. Of the 63 patients in whom frozen section was not performed, five already had a cytologically proven malignancy.

In 10/79 (13%) patients, the result was negative for malignancy even though DTC was found on final pathological analysis. In 2/79 (0.3%) patients, frozen section analysis was indecisive with regard to the nature of the lesion although the final report diagnosed DTC. In the remaining 67 patients (47%), frozen section yielded DTC during the surgical procedure.

Of these 79 patients with frozen section, 12 already had prior FNB findings suspicious of malignancy, leaving 67 patients in whom the frozen section revealed malignancy for the first time. In these 67 patients without positive FNB, 2 had an inconclusive prior FNB while 9 patients had a negative one. In all, in 56/142 (37%) patients, DTC was detected de novo while performing surgery.

In 24/142 (17%) patients, a hemithyroidectomy was performed in the first instance; the remaining patients underwent a total thyroidectomy in a single surgical procedure. In 12/24 patients who thus required a completion thyroidectomy no frozen section was done during primary surgery. Two patients who had a positive frozen section nonetheless initially underwent a hemithyroidectomy due to a loss-of-signal in intraoperative neuromonitoring; a completion thyroidectomy was performed upon verification of adequate vocal cord function. In the remaining 10/24 patients, frozen section was negative and a DTC lesion was only discovered on definitive pathological analysis.

### Outcome

During the comparatively short follow-up interval available to us, 82/142 patients were followed closer to home in peripheral practices and did not return to our center for follow-up. Of the remaining patients, 44/142 patients were classified as “in remission.” Of the 17 patients with positive FNB, thus having the diagnosis before performing surgery, 7 (41%) were in remission, 3 (18%) were not and in 7 (41%) cases no data was available. In comparison, 20/56 (36%) patients with the intraoperative diagnosis of DTC via frozen section were in remission, 7 (12%) were not in remission, and in 29 (52%) patients no data were available. Interestingly, of the 69 patients in whom DTC was only diagnosed postoperatively, 17 (25%) were in remission whereas 6 (9%) were not, and in 46 (66%) patients no data were present. Details of the outcome at the time of data collection are given in Table 6.

**Table 6:** Outcome regarding time of diagnosis

	Remission yes	Remission no	No data available	
Preoperative diagnosis	7	3	7	17
Intraoperative diagnosis	20	7	29	56
Diagnosis in final pathological report	17	6	46	69
	44	16	82	142

## Discussion

The present results show that in a large part of patients who ended up being referred to our center for subsequent differentiated thyroid cancer management the perioperative diagnostic algorithm leaves room for improvement. DTC was detected in only 12% patients before surgery, in only 37% while performing surgery.

FNB was performed just in a small group of patients. Comparing our results published by other groups, however they do not compare unfavorably. The rate of preoperative FNB is quite similar to that reported in another series (19% in the present study vs. 21% in a series of patients with uninodular goiter [2, 3]). In German guidelines, FNB is recommended unequivocally before surgery of benign thyroid diseases in case of the presence of nodules suspicious for malignancy [5]. However, as the present study shows, this recommendation in most cases was not followed, even though it is well known and widely accepted that FNB is able to verify malignancy on the one hand and help avoid unnecessary thyroid surgery on the other [6–8].

Comparing our data to others, e.g., data from Italy, another European and formerly iodine-deficient country, about 57% of new thyroid cancer cases had a diagnosis before performing surgery [9]. Although there is no clear explanation why this rate is considerably lower in Germany, the present comparison demonstrates the particular potential for improvement. The need for such improvement becomes all the more prominent when comparing the outcome data of those with pre- or intraoperatively diagnosed DTC and those with a postoperative diagnosis: remission rates seem to be markedly lower in those with postoperative initial diagnosis, although a meaningful statistical analysis on this particular point was precluded due to the high number of missing data.

Furthermore, only slightly more than half of our patients received intraoperative diagnostics via frozen section, in spite of the recommendation given in German national surgical guidelines [5]. The reason for deviating from the guidelines was not documented explicitly in most cases. Therefore, it is not clear how many patients, if any, could have e.g., been spared a two-step thyroidectomy. Although some newer studies retrospectively reviewing surgery show that there were no significant differences in the risk of hypocalcaemia (transient and permanent), unilateral recurrent laryngeal nerve palsy (transient and permanent) and/or hematoma after a second completion thyroidectomy compared to a one-step procedure [10, 11], general surgical and anesthesia risks can still be minimized by aiming to perform a one-step approach. Still, the issue of frozen section is subject to debate and some international guidelines no longer advocate its routine use [12].

Mayooran et al. showed a clear correlation between FNB and frozen section with the definitive histology of thyroid cancer and concluded that “FNAC [abbreviation of: fine-needle aspiration cytology] is considered as the best modality to triage the thyroid nodule preoperatively,” and that “the intraoperative frozen sections are helpful to perform a one-stage operation for suspicious thyroid lesions” [13]. It is therefore unfortunate to notice that the present investigation of clinical practice in perioperative diagnostics in thyroid cancer patients reveals quite similar numbers as in the aforementioned previous studies [2]. Therefore, further efforts will be needed to further move daily clinical practice to routine application of an optimal combination of diagnostic procedures before and during thyroid surgery.

As with any retrospective study, the present study has a number of weaknesses inherent to such a design. First, as we have investigated a referral center situation, the present study likely underestimates the extent of thyroid cancers found as incidentalomas on surgical treatment of supposedly benign thyroid disease, as solitary microcarcinomas, not requiring I-131 therapy, will not be regularly referred to our center. Furthermore, the retrospective nature of data collection, relying on written reports sent to us for referral purposes, does often not offer detailed explanations for not performing a particular diagnostic test such as intraoperative frozen section analysis. Thus, it is conceivable that non-documented clinical factors may have provided a pragmatic, practical reason for not performing such a test. The latter argument does, however, not invalidate the statistics of the present study, as regardless of the reason, the test was not performed in spite of the presence of both a valid indication and clear potential clinical benefit for the patient.

For clinical practice, the present results must be regarded as a renewed wake-up call: DTC is diagnosed in only 12% of patients before (via FNB) and in 47% (via frozen section) of patients during surgery, thus indicating a need for continuing striving for improvement of thyroid diagnostics. Thus, quality of patients’ lives can be increased and costs of care can potentially be decreased by a reduction in the number of unnecessary primary and secondary surgical procedures.

Furthermore, this investigation in a tertiary referral center may provide initial hints toward explaining central differences of opinion regarding initial DTC treatment between experts in the field of thyroid cancer management. In some high-level expert centers in the world, patients will likely on average have had more complete perioperative assessment, leading to more radical surgery with oncological intent. In our practice, due to insufficient perioperative diagnostics, many patients have not undergone surgery with oncological intent, thus limiting radicality of thyroid surgery and potentially necessitating postoperative radioiodine therapy. Further studies into the precise differences in perioperative diagnostics between centers will be necessary to confirm this hypothesis.

## Conclusion

There is a room for improvement in perioperative diagnostics surrounding thyroid surgery. More frequent use of FNB and frozen section analysis might be able to decrease the number of unnecessary thyroid surgeries and increase the frequency of DTC patients operated upon with oncologically radical intent. Furthermore, differences in the use of perioperative diagnostics might contribute to discordance between experts in the field of DTC treatment.

## Data Availability

Data will be made available upon request.
